# Study on the Catalytic Oxidation of Toluene Using CeO_2_@S-AZMB Prepared from Spent Zn-Mn Batteries

**DOI:** 10.3390/molecules29030616

**Published:** 2024-01-27

**Authors:** Yu Zou, Huan Du, Zhong Zhao, Zhuozhi Wang

**Affiliations:** 1 Sichuan Water Conservancy College, School of Resource and Environmental Engineering, Chengdu 611231, China; 2 Tianjin Key Laboratory of Clean Energy and Pollutant Control, School of Energy and Environmental Engineering, Hebei University of Technology, Tianjin 300401, China

**Keywords:** CeO_2_, spent alkaline Zn-Mn battery, thermocatalysis, toluene

## Abstract

The recycling and utilization of waste alkaline zinc manganese batteries (S-AZMB) has always been a focus of attention in the fields of environment and energy. However, current research mostly focuses on the recycling of purified materials, while neglecting the direct reuse of waste batteries. Here, we propose a new concept of preparing thermal catalysts by combining unpurified S-AZMB with CeO_2_ by means of ball milling. A series of characterizations and experiments have confirmed that the combination with S-AZMB not only enhances the thermal catalytic activity of CeO_2_ but also significantly enhances the concentration of surface oxygen vacancies. In the toluene removal experiment, the temperature (T90) at 90% toluene conversions of CeO_2_@S-AZMB was 180 °C, lower than the 220 °C for CeO_2_. More noteworthy is that this S-AZMB-based thermal catalyst can maintain a good structure and thermal catalytic stability in cyclic catalysis.

## 1. Introduction

As a typical indoor and industrial volatile organic compound (VOC) pollutant, the removal of toluene has become a research hotspot in the field of VOC treatment. Catalytic oxidation technology has been widely used in the removal of toluene due to its higher purification efficiency. Catalysts are the core of thermal catalysis technology. At present, the catalysts that are widely studied mainly include noble metal catalysts and metal oxide catalysts. Metal oxide catalysts are widely used because of their stable catalytic activity, high thermal stability, high availability, and low price [1,2,3,4]. A large number of studies have shown that both MnOx and CeO_2_ have high thermal catalytic activity, and the combination of MnOx and CeO_2_ can form a Mn–Ce composite catalyst with higher activity [5,6,7,8]. In recent years, Mn–Ce composite catalysts have attracted extensive attention from researchers due to their high catalytic activity, and a large number of studies have been carried out on the disadvantages of Mn–Ce catalyst particles such as their easy agglomeration and uncontrollable morphology. These previous studies showed that the morphology of these catalysts can be changed by controlling the preparation method and conditions of the catalyst and that microsphere catalysts have good catalytic activity among the many morphologies [9,10,11,12,13].

Waste zinc manganese batteries contain a large amount of manganese and zinc, and studies have shown that catalysts prepared from waste zinc manganese batteries have good catalytic activity [14,15,16]. Gallegos et al. [17,18] used a biological hydrometallurgy process to recover manganese in the form of oxides from waste alkaline batteries and studied the catalytic performance of the samples for ethanol and heptane. The results showed that the catalytic performance of MnOx recovered from waste batteries was higher than that of MnOx made in the laboratory. Ethanol was completely oxidized at 200 °C, while heptane was completely oxidized at 400 °C. The better performance of the MnOx catalyst could be due to the higher Mn/Zn ratio and the absence of a crystallized ZnO phase. Hoseini et al. [19] recovered manganese oxide powder from waste alkaline batteries, impregnated manganese oxide onto alumina to synthesize composite catalysts, and applied them to the catalytic oxidation of mixtures of benzene, toluene, and xylene. The results indicate that the manganese oxide catalyst prepared from waste batteries has good catalytic performance for toluene and benzene. Kim et al. [20] treated the internal powder of waste zinc manganese batteries with 0.1 N of sulfuric acid solution to obtain a black mass-based catalyst, and prepared Pd/SBM (the black mass of spent Zn/Mn alkaline battery) catalysts using the impregnation method, applying the catalytic oxidation of benzene, toluene, and o-xylene (BTX). The results indicate that Pd/SBM has good thermal catalytic activity, and the complete oxidation temperatures of BTX are 310, 260, and 250 °C, respectively. In summary, catalysts prepared from waste zinc manganese batteries exhibit high thermal catalytic activity towards VOCs, and the performance of manganese-based catalysts prepared from waste zinc manganese batteries is superior to that of pure manganese-based catalysts prepared under the same conditions.

At present, most studies on the process of recycling waste zinc manganese batteries to prepare catalysts introduce a large volume of an acid and alkali solution, resulting in a large amount of waste liquid and causing secondary pollution. In addition, research mainly focuses on the recycling and utilization of one or two specific useful substances in waste zinc manganese battery core powder. Other substances have not been effectively treated, making it impossible to fully recycle and solve the problem of waste battery pollution. Therefore, it is imperative to develop a catalyst preparation method without secondary pollution and to achieve the complete recovery of waste zinc manganese batteries, which has high research significance and practical value.

In order to fully study the application prospects of catalysts for purifying VOCs in the preparation of waste zinc manganese batteries, and to better align with the development trend of VOC treatment technology, this paper conducted research on the preparation of thermal catalysts by using spent alkaline Zn-Mn batteries (S-AZMB) as raw materials. Using S-AZMB as the raw material, a composite with self-made microsphere-shaped CeO_2_ was created using the ball milling method to form CeO_2_@S-AZMB, and we tested the catalytic oxidation activity of the catalyst with toluene as the target pollutant. At the same time, the widely used hydrothermal synthesis method was also used to prepare a Ce-S-AZMB catalyst, which was compared with the catalyst prepared by means of the ball milling method. The purpose was to explore more effective and efficient catalyst preparation methods, providing better ideas and methods for the recovery of waste zinc manganese batteries and the preparation of catalysts for the degradation of VOCs.

## 2. Results and Discussion

### 2.1. Phase and Microstructure

**Figure 1:**
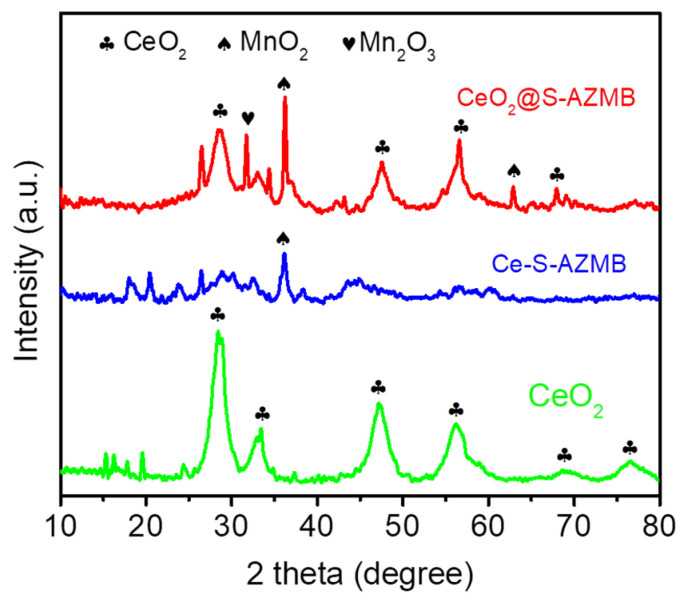
XRD patterns of CeO_2_@S-AZMB, Ce-S-AZMB, and CeO_2_.

The phase structure of the catalyst was characterized by X-ray diffraction (XRD), and the results are shown in Figure 1. The main diffraction peak positions of the CeO_2_ catalyst (which was synthesized in our laboratory) were 28.36°, 33.47°, 47.38°, 59.17°, 69.24°, and 77.31°, respectively. These diffraction peaks corresponded to the (111), (200), (220), (311), (222), and (400) crystal planes of the CeO_2_ cubic fluorite structure in the Fm-3m space group, respectively [21]. It can be seen from the figure that the diffraction peak position of the corresponding CeO_2_ from CeO_2_@S-AZMB was basically the same as that of the catalyst CeO_2_, indicating that the crystal structure of CeO_2_ was not changed after ball milling. An obvious MnO_2_ diffraction peak was found in the catalyst CeO_2_@S-AZMB. In the catalyst Ce-S-AZMB, no obvious CeO_2_ diffraction peak was found, indicating that the addition of S-AZMB hindered the hydrothermal synthesis of CeO_2_. Analyzing the reason, it is possible that CeO_2_ was reduced to other substances by the carbon from the S-AZMB under hydrothermal conditions.

**Figure 2:**
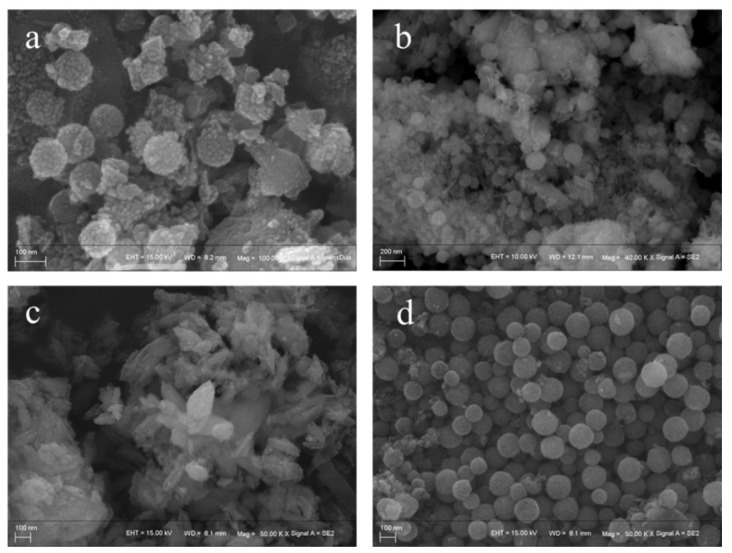
SEM image of (**a**,**b**) CeO_2_@S-AZMB, (**c**) Ce-S-AZMB, and (**d**) CeO_2_.

The specific surface area and pore size distribution of the different catalysts were characterized via BET, and the results are shown in Figure S1a. It can be seen from Figure 2a that all catalysts present with obvious type IV isotherm H3 hysteresis loops, indicating that all samples are mesoporous materials [22]. Figure S1b shows the pore size distribution of the catalysts. The pore size of CeO_2_ was mainly distributed around 4 nm and 30 nm. When combined with S-AZMB, the pore size of CeO_2_ was occupied and the pore volume decreased. Compared with Ce-S-AZMB, CeO_2,_ and S-AZMB in CeO_2_@S-AZMB were more evenly distributed. The specific surface area, pore volume, and average pore size of the catalysts are shown in Table 1. The specific surface area of the self-made nanospherical CeO_2_ was 143.99 m^2^/g. The specific surface area of the catalyst CeO_2_@S-AZMB was only 44.04 m^2^/g after ball milling with waste battery core powder. The specific surface area of CeO_2_-S-AZMB prepared by means of the hydrothermal synthesis method was 92.93 m^2^/g, indicating that the hydrothermal synthesis method was more conducive to the synthesis of catalysts with a large specific surface area than the ball milling method. However, the distribution of active components in CeO_2_@S-AZMB was more uniform, which was conducive to the interaction between active components and improved the catalytic activity. Compared with CeO_2_, the specific surface area of CeO_2_@S-AZMB decreases significantly. Research experience has shown that the decrease in specific surface area in thermal catalytic reactions can lead to a decrease in catalytic performance.

The morphology of the catalyst was characterized by scanning electron microscopy (SEM), and the results are shown in Figure 2. Figure 2d shows the morphology of nanospherical CeO_2_. It can be seen that spherical CeO_2_ with a diameter of about 100 nm was successfully prepared. Figure 2a,b shows the morphology of CeO_2_@S-AZMB. From the morphology, it can be observed that ball milling did not change the spherical structure of CeO_2_, and the spherical CeO_2_ and S-AZMB were uniformly mixed, which was consistent with the BET pore size distribution analysis results. It can be seen from Figure 2c that the direct hydrothermal reaction of a cerium-based precursor with S-AZMB failed to produce spherical CeO_2_, and the product had a rhombic irregular structure.

### 2.2. Oxidation-Reduction Capacity

**Figure 3:**
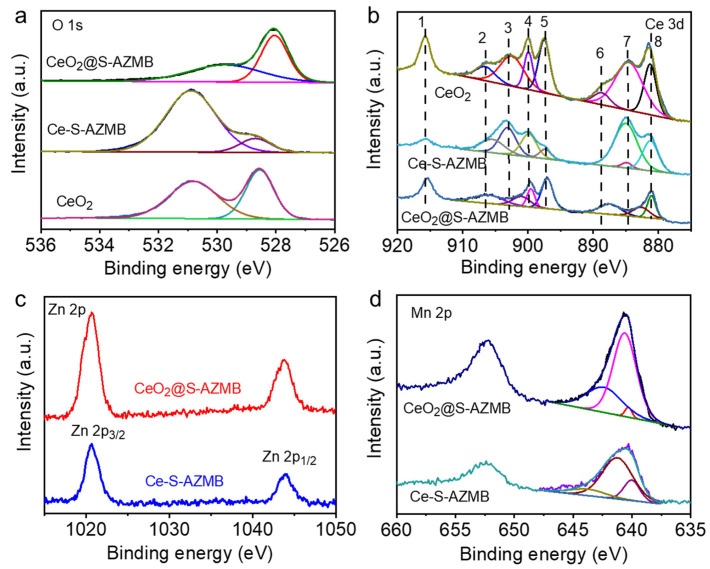
(**a**) O 1s spectra, (**b**) Ce 3d spectra of CeO_2_@S-AZMB, Ce-S-AZMB, and CeO_2_, (**c**) Zn 2p spectra, (**d**) Mn 2p spectra of CeO_2_@S-AZMB and Ce-S-AZMB.

The surface composition and chemical state of the catalyst were determined and analyzed by means of the X-ray photoelectron spectroscopy (XPS) characterization method, and the C 1s peak (BE = 284.8 eV) was used as the standard for binding energy calibration. Figure 3a shows the high-resolution XPS spectrum of O 1s. The position of the characteristic peak of CeO_2_@S-AZMB shifted significantly in the direction of lower binding energy, decreasing the activation energy of the reaction, which was conducive to the reaction [23]. Figure 3b shows the high-resolution XPS spectrum of Ce 3d, which can be divided into seven peaks, of which 1, 2, 3, and 4 are the characteristic peaks of Ce^3+^ and 5, 6, and 7 are the characteristic peaks of Ce^4+^. A large number of studies have shown that the greater the Ce^3+^ content in the catalyst, the higher the concentration of oxygen vacancies on its surface [24,25,26]. According to our calculations, the Ce^3+^/(Ce^3+^ + Ce^4+^) content of the three catalysts was 45.65% (CeO_2_@S-AZMB), 18.15% (Ce-S-AZMB), and 16.5% (CeO_2_), from which we could see that the Ce^3+^ content of CeO_2_@S-AZMB was the highest, and it could be inferred that the concentration of oxygen vacancies on its surface was the highest. The higher the surface oxygen vacancy concentration, the stronger the oxidation performance of the catalyst [27,28], indicating that the CeO_2_@S-AZMB had the highest catalytic oxidation activity. Figure 3d shows the high-resolution XPS spectrum of Mn 2p. Three peaks at 640.7, 641.7, and 642.9 eV can be obtained by means of peak division, which correspond to Mn^2+^, Mn^3+^, and Mn^4+^ [29,30]. The higher the content of Mn^4+^, the more favorable it is for the catalytic oxidation of VOCs. The content of Mn^4+^ of CeO_2_@S-AZMB is much higher than that of Ce-S-AZMB. The higher the content of Mn^4+^, the stronger the oxidation performance of the catalyst, further confirming that CeO_2_@S-AZMB had high catalytic oxidation activity.

**Figure 4:**
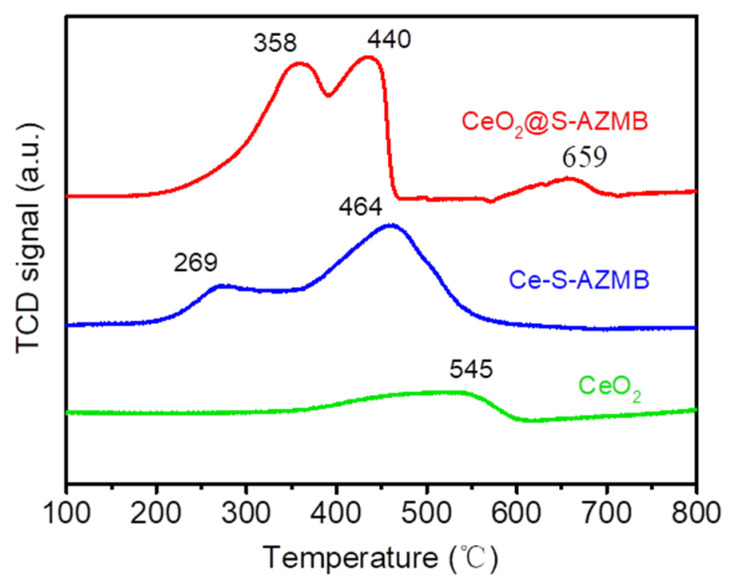
H_2_-TPR profiles of CeO_2_@S-AZMB, Ce-S-AZMB, and CeO_2_.

A H_2_-TPR spectrogram was used to characterize the active oxygen content of the catalyst, and the results are shown in Figure 4. There were three characteristic peaks at 358 °C, 440 °C, and 659 °C, representing the hydrogen consumed by the active oxygen released during the oxidation-reduction reaction on the catalyst surface. The higher the consumption of H_2_, the higher the active oxygen content of the catalyst, contributing to the increase in the oxidation capacity [23,31]. By comparing the integral values of the hydrogen consumption peaks of the three catalysts, the hydrogen consumption of CeO_2_@S-AZMB was determined to be the highest, much higher than that of spherical CeO_2_. It can be seen that the oxidation ability and thermal catalytic oxidation activity of CeO_2_ were enhanced after mixing with S-AZMB.

**Figure 5:**
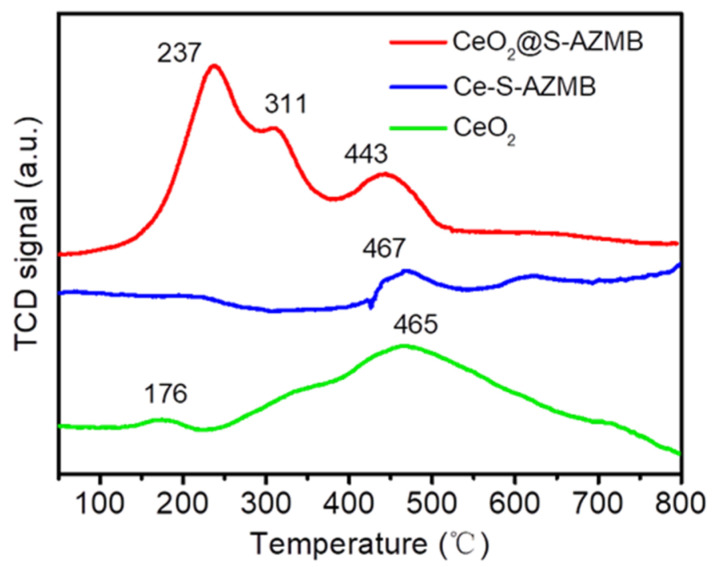
O_2_-TPD profiles of CeO_2_@S-AZMB, Ce-S-AZMB, and CeO_2_.

The distribution of oxygen species on the catalyst surface was characterized by means of the O_2_-TPD method, and the results are shown in Figure 5. Generally, the oxygen species of metal oxides mainly included surface active oxygen, surface lattice oxygen, and bulk lattice oxygen, of which the surface active oxygen was most easily desorbed from the catalyst surface. The desorption peak below 500 °C belongs to the surface active oxygen species, the desorption peak at 500 °C–700 °C belongs to the surface lattice oxygen species, and the desorption peak above 700 °C belongs to the bulk lattice oxygen species [32,33]. It can be clearly observed that CeO_2_@S-AZMB had strong desorption peaks at 237 °C, 311 °C, and 433 °C, and the intensity was significantly higher than that of CeO_2_ at low temperatures, proving that the catalyst CeO_2_@S-AZMB had more surface active oxygen, which was consistent with the XPS analysis results and indicated that CeO_2_@S-AZMB has higher thermal catalytic oxidation activity.

**Figure 6:**
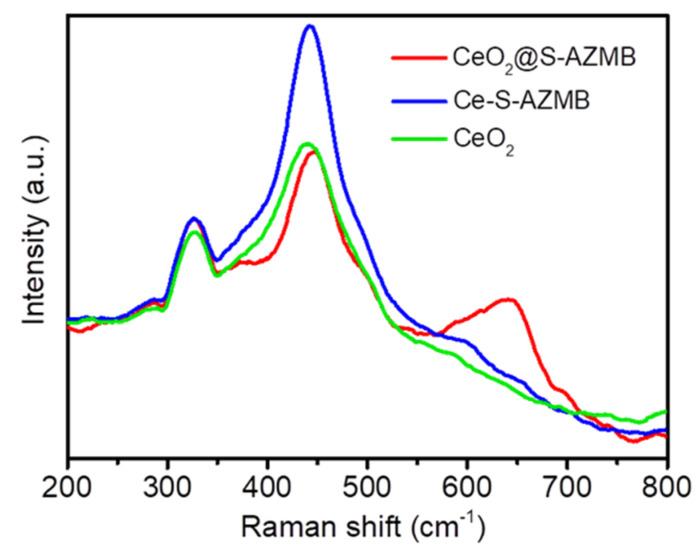
Raman spectra of CeO_2_@S-AZMB, Ce-S-AZMB, and CeO_2_.

The structural difference and oxygen vacancy information of the catalyst were analyzed and measured by means of Raman spectroscopy. The experimental results are shown in Figure 6. It can be clearly seen from the figure that the characteristic peak patterns of the three samples at 324 and 421 cm^−1^ were similar, and there was no obvious shift, indicating that the structure of CeO_2_ had not changed significantly. In addition, it was also observed that the intensity of the F2g symmetric stretching vibration peak of the three catalysts was different. The stronger the peak intensity was, the better the long-range order of CeO_2_ was, and the higher the crystallinity was. However, it also meant that the higher the disorder of the Ce-O bond was, the more likely it was to form oxygen vacancies [27,28]. As can be seen from the figure, CeO_2_@S-AZMB F2g symmetric stretching vibration peak intensity was the lowest, proving it could more easily form surface oxygen vacancies and had a higher surface oxygen vacancy concentration, which was consistent with the analysis results of XPS and O_2_-TPD. The increase in surface oxygen vacancy concentration was beneficial to the increase in oxygen adsorption on the catalyst surface, thus improving the thermal catalytic oxidation activity of the catalyst. The peak at 643.5 cm^−1^ of CeO_2_@S-AZMB corresponded to the symmetric tensile vibration of v2 (MnO) in the MnO6 octahedron.

**Figure 7:**
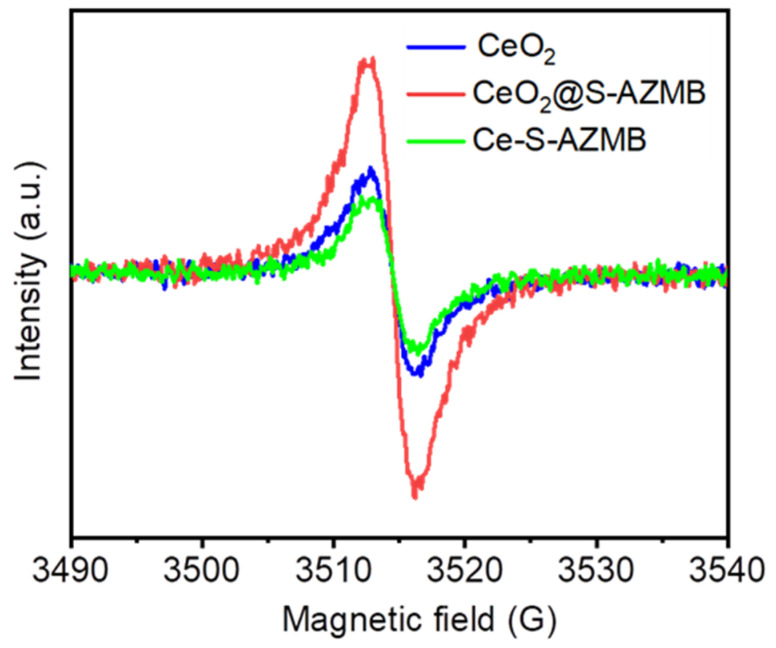
ESR spectra for oxygen vacancies of CeO_2_, CeO_2_@S-AZMB, and Ce-S-AZMB.

In order to more directly prove the presence of oxygen vacancies in the catalyst, ESR technology was used to characterize the oxygen vacancy formation in the catalyst, and the results are shown in Figure 7. It can be clearly observed from the figure that the peak intensity of oxygen vacancy diffraction of CeO_2_@S-AZMB was the highest, proving that the surface oxygen vacancy concentration of CeO_2_@S-AZMB was the highest, which was consistent with the results of XPS and Raman characterization. This also more directly proved that CeO_2_@S-AZMB had the highest surface oxygen vacancy concentration, which could further imply that the thermal catalytic oxidation activity of CeO_2_@S-AZMB was the highest.

### 2.3. Thermal Catalytic Activity and Stability

**Figure 8:**
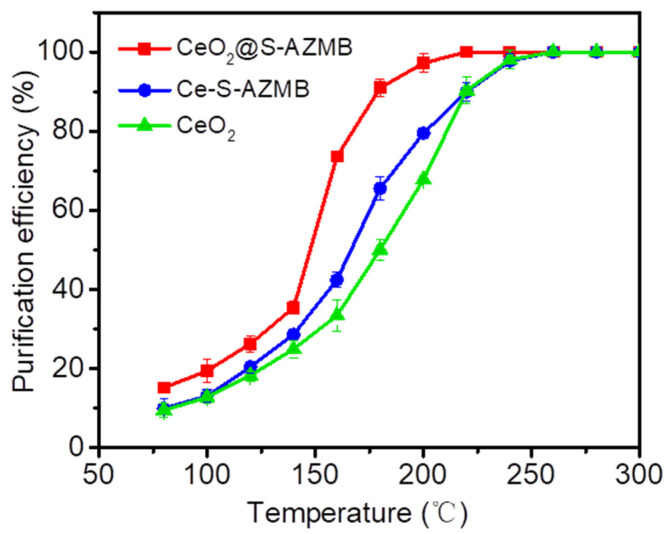
Catalytic oxidation efficiency of toluene with CeO_2_@S-AZMB, Ce-S-AZMB, and CeO_2_. (Toluene concentration: 400 ppm; WHSV: 120,000 mL g^−1^ h^−1^; catalyst amount: 200 mg).

The thermal catalytic oxidation performance of catalysts was evaluated using toluene as the pollutant. The thermal catalytic oxidation efficiency of different catalysts for toluene is shown in Figure 8. It can be observed in the figure that the catalytic oxidation efficiency of p-toluene of CeO_2_@S-AZMB is higher than that of CeO_2_ and Ce-S-AZMB. The T90 (temperature required when the catalytic oxidation efficiency reaches 90%) of CeO_2_@S-AZMB was 180 °C, lower than 220 °C of CeO_2_ and Ce-S-AZMB. The complete catalytic oxidation of toluene of CeO_2_@S-AZMB was achieved at 220 °C, which is 40 °C lower than that of CeO_2_. The thermal catalytic activity of CeO_2_@S-AZMB was higher than that of CeO_2_ and Ce-S-AZMB. The reaction of toluene on the surface of the metal oxide catalysts followed the Mars–Van Krevelen mechanism. Therefore, the activation of oxygen on the surface of the catalysts was an important factor in the thermal catalytic process of toluene. The stronger the oxidation ability of the catalyst, the higher the thermal catalytic performance was. It can be seen from the experimental results of the catalytic oxidation of toluene that CeO_2_@S-AZMB had the best catalytic oxidation degradation efficiency of toluene, which was consistent with the previous XPS, Raman, H_2_-TPR, EPR, and other characterization results. After mixing CeO_2_ with S-AZMB, the new composite catalyst had a higher surface oxygen vacancy concentration, a stronger catalytic reduction performance, and a higher catalytic oxidation degradation efficiency.

**Figure 9:**
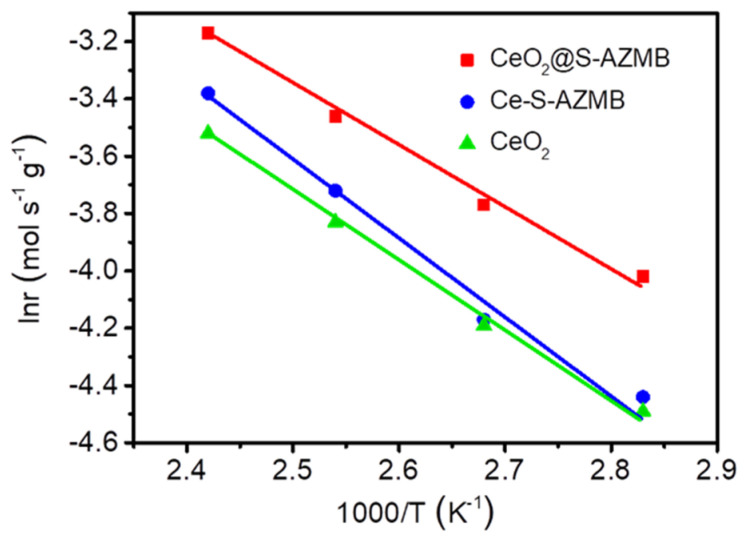
Arrhenius plots for toluene catalytic oxidation with CeO_2_@S-AZMB, Ce-S-AZMB, and CeO_2_.

In order to further study the activity difference between catalysts, the Arrhenius point of each sample at a low temperature (when the toluene removal efficiency was less than 20%) was calculated and linearly fitted, and the results are shown in Figure 9. The slope of the fitting line in the figure corresponded to the apparent activation energy of the sample in the reaction. The apparent activation energy usually represents the difference between the average energy required for activating molecules and the average energy of all molecules. The lower the apparent activation energy, the more easily a reaction will occur, indicating the higher activity of the catalyst [28,29]. The apparent activation energies of CeO_2_@S-AZMB, Ce-S-AZMB, and CeO_2_ were 39.17, 49.85, and 44.51 kJmol^−1^, respectively. The lowest apparent activation energy of the CeO_2_@S-AZMB reflects that the catalyst modified by ball milling with waste zinc manganese batteries was more conducive to the adsorption and activation of reactant molecules on its surface, and the catalytic oxidation of toluene was more likely to occur. According to the previous characterization results, although CeO_2_@S-AZMB has the smallest specific surface area, it has the highest toluene removal efficiency, which can be attributed to the abundant surface oxygen vacancies and ultra-high oxidation-reduction ability of CeO_2_@S-AZMB.

**Figure 10:**
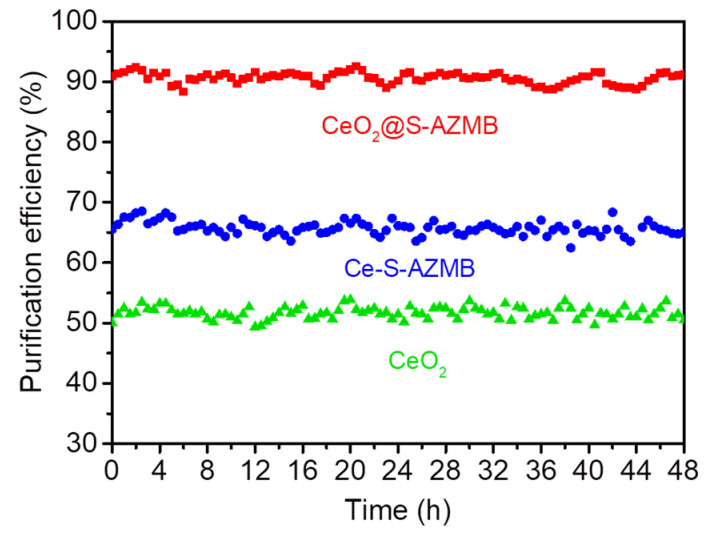
The stability of the catalyst for continuous reaction at 180 °C for 48 h. (Reaction temperature: 180 °C; toluene concentration: 400 ppm; WHSV: 120,000 mL g^−1^ h^−1^; catalyst amount: 200 mg).

In order to determine the stability and reusability of the catalyst, continuous and cyclic experiments were carried out on the catalyst, respectively. The continuous test was carried out at 180 °C, the catalyst was continuously reacted for 48 h, and a sample was taken every 30 min to test the purification efficiency of toluene. The experimental results are shown in Figure 10. It can be seen from the figure that the stability of the three catalysts was very good. After 48 h of continuous reaction, the purification efficiency of the catalyst for toluene experienced little change. We found that spherical CeO_2_ prepared by means of the hydrothermal method had good stability, and the CeO_2_@S-AZMB modified with ball-milled waste zinc manganese battery core powder not only had greatly improved catalytic activity but also maintained good stability.

**Figure 11:**
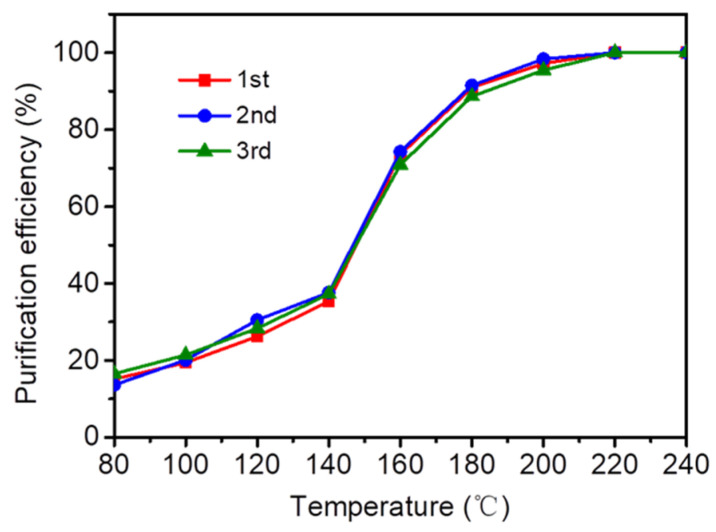
The reusability of toluene removal of CeO_2_@S-AZMB. (Toluene concentration: 400 ppm; WHSV: 120,000 mL g^−1^ h^−1^; catalyst amount: 200 mg).

In general, during the cooling process after the completion of the thermal catalytic reaction, the catalyst would retain some intermediate products and experience carbon deposition on its surface due to the incomplete degradation of pollutants. This carbon deposition can lead to a reduction in catalyst activity and even poisoning and deactivation. Therefore, it was of great significance to test the cyclic service life of the catalyst for the practical application of the catalyst. The catalyst’s function was regarded as a cycle from the beginning of heating up to the complete degradation of toluene and then to room temperature. Three consecutive cycle experiments were carried out to evaluate CeO_2_@S-AZMB, and the experimental results are shown in Figure 11. It could be observed that the results of the three-cycle experiments were similar. Each time, the purification efficiency of toluene reached 90% at 180 °C, and the completed removal of toluene could be achieved at 220 °C. This not only proved that it had good recycling performance, but also demonstrated that toluene could be completely converted into CO_2_ and H_2_O at 240 °C, and there was almost no by-product at the end of the reaction, reducing the impact of carbon deposition.

**Figure 12:**
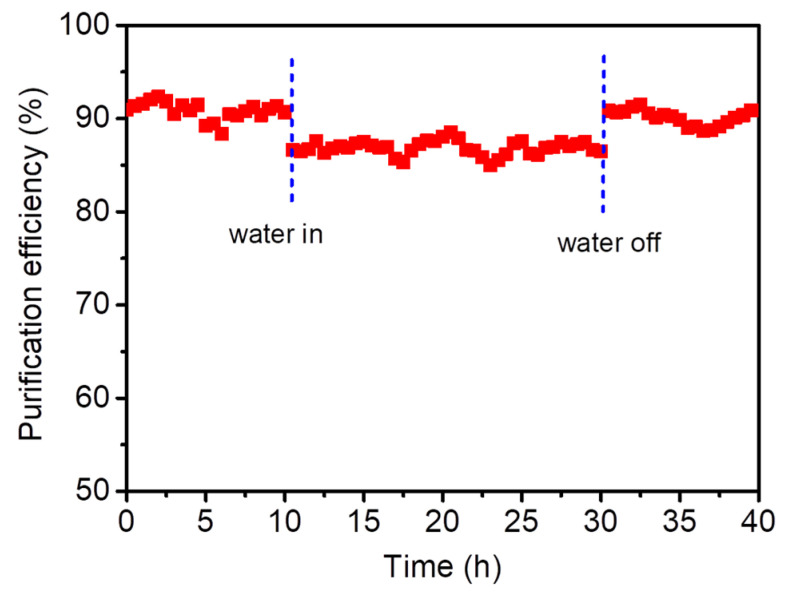
The water resistance of catalyst. (Reaction temperature: 180 °C; toluene concentration: 400 ppm; WHSV: 120,000 mL g^−1^ h^−1^; catalyst amount: 200 mg; water vapor content: 5 vol%).

In the catalytic oxidation of toluene, H_2_O usually affects the activity of the catalyst. When the concentration of H_2_O is too high, it will even cause the deactivation of the catalyst. Therefore, it is also important to test the water resistance of the catalyst. Taking CeO_2_@S-AZMB as the research object, water vapor was introduced into the catalytic oxidation reaction at 180 °C, and the reaction was stopped in the presence of water for 20 h. The purification effect of toluene in the presence or absence of water vapor is shown in Figure 12. It can be seen from the figure that, with the addition of water vapor, the purification efficiency of toluene of CeO_2_@S-AZMB decreased from about 90% to about 86%, and remained stable in the reaction lasting 20 h. When the application of water vapor ceased, the purification efficiency of toluene of CeO_2_@S-AZMB recovered to about 90% and remained relatively stable. The experimental results indicate that although the presence of water vapor can have a certain inhibitory effect on the activity of the catalyst, the effect was not significant, and CeO_2_@S-AZMB had good water resistance. Some studies have shown that water vapor will not only engage in competitive adsorption with toluene on the catalyst surface but also lead to the reduction in the catalyst’s active oxygen capacity, thereby reducing the catalyst’s catalytic activity and affecting the purification efficiency [34]. Combined with the characterization results, although the specific surface area of CeO_2_@S-AZMB was low, the rich surface oxygen vacancies provide more reactive sites and stronger oxidation-reduction ability, providing the reason for its good water resistance.

## 3. Materials and Methods

### 3.1. Materials and Fabrication

All reagents used in this work were of analytical grade. Spent alkaline Zn-Mn batteries powder were obtained by a simple process. The spent alkaline Zn-Mn batteries (NANFU Battery Plant, Nanping, China) were collected in our daily lives and the internal residues were collected after the battery metal shell was mechanically removed. Then, the internal residues were washed repeatedly with deionized water and dried at 105 °C for 10 h. At last, the product was obtained and grinded into powder, labeled as S-AZMB.

Nano-spherical CeO_2_ was prepared. First, Ce(NO_3_)_3_·6H_2_O (4 g) and deionized water (4 mL) were mixed into a beaker and were stirred under magnetic force until they were completely dissolved. Then, glycol (120 mL) was added to the solution and continued stirring for 5 min. Finally, propionic acid (4 mL) was also injected into the solution, fully stirred for 30 min, until the solution became viscous and suspended. The fully stirred solution was put into a 200 mL hydrothermal reactor and reacted for 12 h at 180 °C. After the reaction, it naturally fell to room temperature. The solid-liquid mixture was centrifuged at 10,000 r/min for 10 min. The centrifuged solid was taken out and washed with water. The solid was washed and centrifuged repeatedly until the centrifuge solution was neutral. Finally, the solid was dried completely at 105 °C. The yellow powder was CeO_2_, named CeO_2_. The preparation method of Ce-S-AZMB is the same as CeO_2_, but S-AZMB (2.378 g) needs to be added during solution stirring.

The waste Zn-Mn battery composite catalyst was prepared by the ball milling method. The self-made nanospheres CeO_2_ (2 g) and S-AZMB (6 g) were added into the ball milling tank. An appropriate amount of anhydrous ethanol was added in, and ball milling at 500 r/min for 6 h. The sample was taken out after ball milling and completely dried at 80 °C. Then the dried sample was put into the muffle furnace and calcined at 200 °C for 4 h in an air atmosphere. The solid obtained after natural cooling was named CeO_2_@S-AZMB.

### 3.2. Characterization

The morphology of the samples was characterized by scanning electron microscopy (Nova Nano SEM 460, FEI, Hillsboro, OR, USA) and transmission electron microscopy (TEM, Talos F200X, FEI). The detailed information of structural phase, chemical composition, and elemental of the samples were determined by X-ray diffraction (Shimadzu XRD-6100, Shimadzu, Kyoto, Tokyo, Cu Kα radiation, λ = 1.5418 Å) and X-ray photoelectron spectroscopy (phi-5700 ESCA, Al Kα X-ray). C 1s peak (BE = 284.8 eV) was used as the standard for binding energy calibration. The specific surface area and pore size distribution of the samples were determined by Brunauer–Emmett–Teller (BET, ASAP 2020 HD88). The redox performance of the samples was determined by O_2_ temperature programmed desorption and H_2_ temperature programmed reduction (O_2_-TPD and H_2_-TPR, Micromeritics AutoChem II 2920). The oxygen vacancy formation of samples was characterized by room-temperature electron spin resonance (EMXplus-6/1) and Raman spectroscopy (Thermo Fischer DXR, Waltham, MA, USA).

### 3.3. Thermal Catalytic Degradation of Toluene

**Figure 13:**
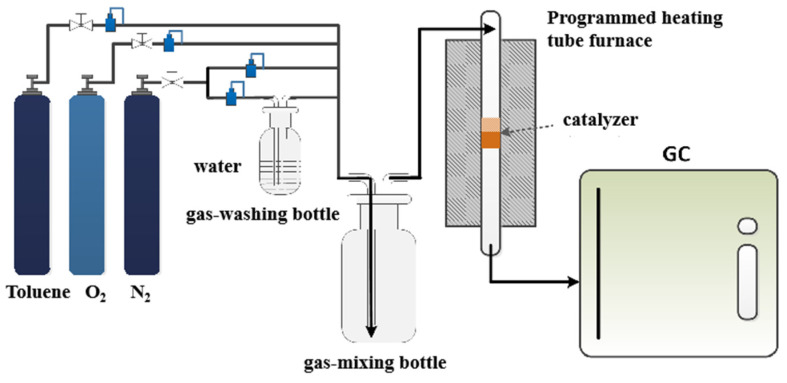
The reaction system of thermocatalysis.

Toluene is selected as the target pollutant, and the fixed bed reactor equipped with a quartz reaction tube is used to test the thermal catalytic oxidation performance of the catalyst. The experimental device is shown in Figure 13. The 400 ppm toluene standard gas is selected as the toluene reaction gas, and oxygen and nitrogen are introduced into the gas mixing bottle at the same time. After the gas mixture is uniform, it is introduced into the thermal catalytic oxidation reaction system. The thermal catalytic oxidation reaction system includes a resistance heating furnace, quartz reaction tube, and temperature controller. Before the reaction, the catalyst shall be pressed into pieces, then ground and crushed, and 40~60 mesh particles shall be screened, 200 mg of catalyst shall be weighed, and the catalyst shall be placed in the quartz reaction tube, and both ends shall be blocked with quartz cotton. After the catalyst is installed, connect the gas circuit, where the oxygen flow is 40 mL/min, the N_2_ flow is 320 mL/min, and the toluene flow is 40 mL/min. Under normal temperatures, the catalyst is subject to toluene adsorption saturation treatment (excluding the influence of catalyst adsorption performance), and the tail gas is collected with a polytetrafluoroethylene sampling bag. After collection, the concentration of toluene is measured by gas chromatography (GC), and a sample is taken every 20 min until the concentration of toluene does not change, and the catalyst reaches adsorption saturation. At this time, the concentration of toluene is taken as the initial concentration of toluene, which is recorded as C_0_. After the catalyst reaches adsorption saturation, raise the temperature of the reaction system. After each temperature point is stabilized for 30 min, sample and measure the concentration of toluene after thermal catalytic oxidation, which is recorded as C_1_.

The purification efficiency of toluene is calculated by the following Formula (1):

X = (1 − C_1_/C_0_) × 100% (1)

At the same time, the temperature (T90) when the toluene purification efficiency is 90% is used to indicate the catalyst activity. In order to further discuss the catalytic oxidation activity of the catalyst, the Arrhenius equation was used to calculate the apparent activation energy of the catalyst at low temperatures:

(2) $$\ln r=-\frac{Ea}{RT}+\ln A$$

where r is the reaction rate of toluene catalytic oxidation (mol/s), Ea is the apparent activation energy of the catalyst (J/mol), R is the gas constant (J/(mol · K)), and T is the reaction temperature (K).

## 4. Conclusions

This article reports on a new method for recovering unpurified S-AZMB by combining it with CeO_2_ to construct a thermal catalytic system. A thermal catalyst was successfully prepared through a simple ball milling and calcination process. Multiple characterizations and experiments have shown that the combination with S-AZMB not only significantly enhances the thermal catalytic activity of CeO_2_ but also effectively increases the content of surface oxygen vacancies. The catalyst has a good thermal catalytic effect on toluene. More importantly, we have confirmed that good thermal catalytic activity, structural stability, and water resistance can be maintained in cyclic reactions. These findings provide new insights into the recycling and reuse of wastebatteries and offer new opportunities for improving the activity of thermal catalysts.

## Figures and Tables

**Table 1:** Structural properties of catalysts.

Samples	Specific Surface Area (m^2^g^−1^)	Pore Volume (cm^3^/g)	Pore Size (nm)
CeO_2_@S-AZMB	44.04	0.12	13.63
Ce-S-AZMB	92.93	0.26	11.02
CeO_2_	143.99	0.29	8.06

## Data Availability

Data are contained within the article and Supplementary Materials.

## Supplementary Materials

The following supporting information can be downloaded at: https://www.mdpi.com/article/10.3390/molecules29030616/s1, Figure S1: (a) N_2_ adsorption/desorption isotherms and (b) Barrett-Joyner-Halenda (BJH) pore size distribution of CeO2@S-AZMB, Ce-S-AZMB and CeO_2_.
