# Supplementary material for: Study on the Catalytic Oxidation of Toluene Using CeO2@S-AZMB Prepared from Spent Zn-Mn Batteries
Source: Molecules. 2024 Jan 27;29(3):616. doi: 10.3390/molecules29030616 (PMC10856000; doi:10.3390/molecules29030616)
Supplement: Supplementary file 1 [file molecules-29-00616-s001.zip › molecules-2822099-supplementary.pdf]

Supplementary data

Study on catalytic oxidation of toluene by CeO_2@S-AZMB prepared from spent Zn-Mn battery

Zou Yu 1, Du Huan 1, Zhao Zhong[2,*], and Wang Zhuozhi [2,*]

1 Sichuan Water Conservancy College, Chengdu 610000, China;

2 Tianjin Key Laboratory of Clean Energy and Pollutant Control, School of Energy and Environmental Engineering, Hebei University of Technology, Tianjin 300401, China;

*Correspondence: Z.Z, qidaiwenjing@163.com；W.Z-Z, 2021115@hebut.edu.cn

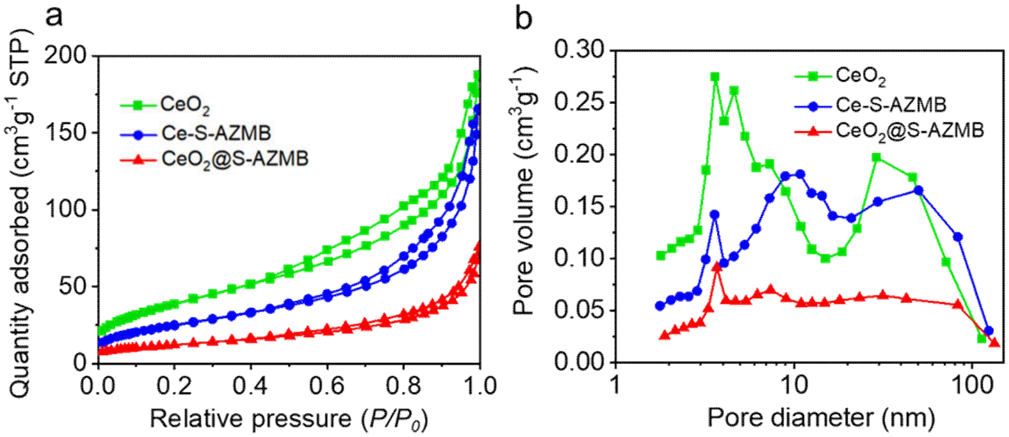

Figure S1. (a) N_2 adsorption/desorption isotherms and (b) Barrett-Joyner-Halenda (BJH) pore size distribution of CeO_2@S-AZMB, Ce-S-AZMB and CeO_2
